# The Perception of Occupation by Hospital Nurses in Poland and Germany in Terms of the Risk of Excessive Stress and Burnout as Well as Possible Coping and Preventive Solutions

**DOI:** 10.3390/ijerph18041797

**Published:** 2021-02-12

**Authors:** Dorota Kwiatkowska-Ciotucha, Urszula Załuska, Cyprian Kozyra

**Affiliations:** 1 Department of Logistics, Wroclaw University of Economics and Business, 53-345 Wroclaw, Poland; dorota.kwiatkowska@ue.wroc.pl (D.K.-C.); urszula.zaluska@ue.wroc.pl (U.Z.); 2 Department of Statistics, Wroclaw University of Economics and Business, 53-345 Wroclaw, Poland

**Keywords:** nursing profession, burnout prevention, working environment, primary research results, comparative analysis

## Abstract

Nurses constitute a professional group exposed to a high risk of stress and occupational burnout. Fewer nurses are recruited every year and the ever higher age of those professionally active is alarming. This article presents the results of international comparative studies from 2018 and 2019 involving Polish and German nurses (747 people) dedicated to the perception of aspects of nurses’ work in terms of the risk of excessive stress and burnout and possible preventive measures. Using a proprietary questionnaire, the authors evaluated differences in the perception of the nursing profession in Poland and Germany, as well as in terms of seniority or decision-making. Next, the relationship between the perception of work specificity and opinions about professional risks and possible preventive measures was evaluated. The analysis used the Mann-Whitney U test and correlation analysis of the questions. Using exploratory factor analysis, the number of dimensions describing the nursing profession was reduced from 16 to four related to (1) workload, (2) job satisfaction, (3) atmosphere in the organisation and (4) sense of control over one’s own work. The results showed statistically significant differences in the perception of professional specificity and risks according to the analysed characteristics.

## 1. Introduction

Contemporary concepts of occupational burnout define this phenomenon as a “disturbed professional relationship” between the individual and the individual’s work and its related requirements [1]. People who perform the so-called helping professions, such as healthcare staff, teachers or caregivers of dependent people [2,3], are particularly vulnerable to such disturbances. The consequences of burnout are very serious both for the individual (demotivation, discouragement, desensitisation, poorer quality of services provided, mental problems) and for the organisation (low efficiency, lack of commitment, creativity, financial losses). This is the result of psychological phenomena causing burnout such as psychophysical and emotional exhaustion, depersonalisation and reduced evaluation of one’s own achievements [4]. In the 21st century, the problem of burnout has been widely recognized. The results of research conducted in the USA and Canada confirmed the universality of this phenomenon and the possibility of its occurrence in various types of occupation [5]. In May 2019, the World Health Organization (WHO) recognized burnout as an “occupational syndrome” which will find its place in the 11th Revision of the International Classification of Diseases: ICD-11) [6] in force from 2022. The extensive literature on the subject shows, however, that despite the proven universality of the burnout phenomenon, it is still taboo. Such an attitude is mostly visible, for example, among academic teachers, where, according to research results, knowledge of burnout is most often obtained from the Internet or observation of colleagues at work, whereas the employee’s formal admission of experiencing burnout is perceived as a sign of weakness [7,8,9].

The situation looks different in the area of healthcare, where due to the constant emotionally burdening contact with disease and suffering burnout is perceived as something normal. In this sector, nurses are particularly at risk [10,11,12] due to direct contact with patients and lower social status in relation to the professional group of physicians. The risks in the area of the nursing profession are particularly important in the context of aging societies or of the state of the current pandemic, observed now for almost a year [13]. A lower number of new nurses recruited and the ever higher average age of those who still work are alarming signals of the need to support this professional group. What is interesting is that the research results indicate lower resistance to stress and burnout in younger age groups. For example, in Ireland [14] younger nurses were more likely to report burnout symptoms (68%) compared to those aged 51 or older (47%). An Australian study delivered similar results, showing that baby boomers reported lower levels of stress and burnout than generations X and Y [15]. Cross-sectional studies conducted simultaneously in many European countries show a growing crisis in the nursing profession. For example, according to studies conducted in 12 countries, more than one in five nurses (ranging from 11% in the Netherlands to 56% in Greece) were dissatisfied with their occupation, and this dissatisfaction was expressed in terms of earnings as well as of educational and development opportunities [16]. The results of this study are worrying, especially when it comes to the percentage of nurses who intend to leave their jobs—this ranged from 19% in the Netherlands to 49% in Greece and Finland. According to the results of studies conducted in a group of Swiss nurses and physicians, on average one in 12 healthcare workers demonstrated severe burnout symptoms, and one in six often thought about leaving the profession. Physical, emotional and mental workload and stress at work were strongly and positively associated with the symptoms of burnout and thoughts of leaving the profession [10]. On the other hand, improving the working conditions of nurses is associated with lower rates of occupational burnout, lower intention to leave the current position and less dissatisfaction with work [17].

The importance of the issues of excessive stress and burnout, as well as the extensive literature on the subject, inspired this comparative study in a group of Polish and German nurses. In order to evaluate possible differences and relationships, a proprietary questionnaire was used, in which respondents were asked about specific aspects of employment and occupational burnout. The study was conducted in 2018–2019, and its aim was to evaluate risks in the area of occupational burnout in the context of professional specificity. It was dedicated to the perception of various aspects of one’s own work in two countries that differ in terms of the objective level of healthcare and, consequently, working conditions. It is worth noting that the study did not measure the phenomenon of occupational burnout among respondents using such well-known scales as Maslach Burnout Inventory (MBI) [1,4] or the Link Burnout Questionnaire (LBQ) [18], but searched for answers on how nurses perceive their own profession in the context of the risk of burnout, the risk of excessive stress, burnout itself, and possible ways of preventing these problems.

The choice of those two countries was not accidental because over the years many Polish nurses have moved to Germany. According to the study results, the situation of nurses in both countries is perceived as bad, but from the perspective of the situation of Polish nurses the attractiveness of better remuneration or working conditions determines their temporary or permanent departure for work. Studies conducted among German nurses indicate, for example, that in the period 1990–2012 their job satisfaction decreased by an average of 7.5%, while the satisfaction of physicians and other healthcare workers increased by 14.4% and 1%, respectively [19]. The professional situation of nurses in various environments seems to be characterised by a perceived imbalance between workload, wages and a high risk of burnout symptoms [20]. The degree of control over one’s own actions is essential in dealing with stress and burnout, and the nurses who feel little control over their lives are more prone to those unfavourable phenomena [21].

Numerous studies conducted among Polish nurses have revealed information about disturbing trends that result in more and more serious shortages of nursing staff, worsening job satisfaction and increasing levels of burnout, which may pose a threat to patient care [22]. It is possible to talk about a crisis, the symptoms of which include the alarmingly high average age of nurses in Poland (52 years), a high percentage of nurses dissatisfied with working conditions [23], a large number of nurses leaving the profession, with a simultaneous increase in the demand for care and nursing services [24]. The research conducted in 2000 and 2014 in the European Union countries showed that Poland ranks sixth from last in terms of the number of nurses per 1000 inhabitants [25]. It is necessary to emphasise that two-thirds of the population of Polish nurses fall into the 41–60 age range, and almost 85% of them are over 40 [26]. The study results indicate statistically significant relationships between occupational burnout and occupational stress among nurses, as well as differentiation in terms of education, age and seniority [27]. It is also possible to notice that people in management positions perceive their situation in a more positive way [24].

The aim of this study was to examine the differences between countries in the perception of risks in the nursing profession and to assess the relationship between the perception of the specificity of work and factors influencing burnout. Two research questions were formulated to structure the analyses.

The first research question: Are there any differences in the perception of the specificity of work in the nursing profession in Poland and Germany, and are there any differences in this area due to specific professional characteristics such as seniority or decision-making (management vs. non-management position)?

The second research question: Are there any relationships between factors such as the perception of work specificity, the assessment of the risk of excessive stress and the possibilities of preventing burnout?

In order to answer these questions, we used a proprietary questionnaire. Selected questions from the research questionnaire that were used in the article are included in Appendix A, Table A1 and Table A2.

## 2. Materials and Methods

### 2.1. Data Collection and Research Sample

The study, which covered nurses working in Polish and German hospitals, was conducted in the period of October 2018–June 2019 using the PAPI method (Paper and Pencil Interview). The staff were employed in hospitals in the Dolnośląskie and Śląskie Voivodeships in the case of Poland and in the city of Neubrandenburg and its vicinity in the case of Germany. We used a proprietary questionnaire which consisted of 12 closed-ended questions with a choice of answers provided. Depending on the nature of the question, the respondents could choose one answer, multiple answers or evaluate the severity of a given phenomenon. In the latter case, the evaluation was made on a scale from 0 to 10. In some questions, it was possible to answer, “Difficult to say”, coded as missing data. The objective of the study was to collect opinions concerning selected aspects of work in the nursing profession and occupational burnout (level of knowledge, degree of risk and prevention possibilities). The study was anonymous. The results were analysed only in an aggregated form and in separate research groups. The submission of the completed questionnaires by the nurses was considered to be their consent to participate in the study.

In total, 747 complete questionnaires were collected—632 from Poland and 115 from Germany. The characteristics of the respondents according to the features included in the respondent’s particulars of the questionnaire form are presented in Table 1.

It is worth noting that the research samples in both countries differed significantly due to all the characteristics shown in Table 1, apart from current job position, which is mainly the result of greater feminization of the nursing profession in Poland and a higher average age of nursing staff in Poland compared to Germany. In the German sample, 26% of the respondents were men, whereas in Poland this share equals 6%. The prevailing age category in Poland was 46–55 years old (42%), while in Germany the distribution of this characteristic was balanced between the first four categories. The different distribution of both samples in the case of age also caused differences in the distribution for the characteristic of seniority. In the German sample, the most common category was seniority of 5–15 years (42%), whereas for the Polish sample this was 25 (53%).

### 2.2. Methods of Data Analysis

We performed basic statistical analyses (frequency, mean and standard deviation calculations). In order to find the answer to the first research question, we used a test of equality of distribution in two groups, which was performed with the non-parametric Mann-Whitney U test due to discrete distribution of item responses. Significance of dependency in cross-tables was tested with the asymptotic chi-squared Pearson test of independence. In order to find the answer to the second question, we analysed the correlation matrix between the questions and searched for the possibility of reducing the dimension of the variables on the basis of their relationship in the correlation matrix. The statements describing job characteristics (items) presented in Table A1 and the questionnaire questions shown in Table A2 express a subjective view (or perception) of nurses towards their own work. They are ad hoc scales for measuring latent characteristics. Therefore, in the case of statements describing the job characteristics, multivariate analyses [28] were carried out based on the correlation matrix between items: reliability analysis with Cronbach’s alpha coefficient, exploratory factor analysis (EFA) with the principal components method and varimax rotation and confirmatory factor analysis (CFA) with the maximum likelihood estimation method. As for performing statistical analyses, we used the statistical software Statistica 12.5 [29] and IBM SPSS 26 [30].

## 3. Results

### 3.1. The Specificity of the Nursing Profession

The perception of the specificity of work in the nursing profession was analysed on the basis of 16 statements (see Table A1 in the Appendix A). The respondents evaluated to what extent a given statement accurately described their work on a scale from 0 to 10, where 0 meant that it did not fit the nature of the work, and 10 that it definitely did. These statements were positive as well as negative. The highest mean value for the entire research sample was noted for the evaluation of work as involving contact with others (9.04). At the same time, for this category there was the smallest differentiation of opinions (standard deviation at the level of 2.10). For the next three statements, the average value exceeded 7. These concerned good fit to the work performed (7.77), too much bureaucracy (7.30) and maintaining work-life balance (7.04). The lowest number of respondents agreed with the statements relating to the willingness to change jobs, stressful relationships with co-workers and superiors. In each of these cases, the mean evaluation was below 4. Relatively significant differentiation was noted in the evaluation of the willingness to change job and take some rest from work (standard deviation at the level of 3.31 and 3.30, respectively). Apart from stating that the work involved contact with other people, a lot of respondents agreed with the statement concerning suitability for the job that one does (standard deviation at the level of 2.45). The significance of differentiation in the opinions of respondents characterised by different features was determined on the basis of the Mann-Whitney U test. We took into consideration characteristics such as country, position held and seniority. The results are presented in Table 2. It should be emphasised that these three binary grouping characteristics are not independent of each other, and that a significant relationship between seniority and the position held was expected (*p* < 0.001). There was no significant relationship between country and the position (*p* = 0.221), but there was a significant relationship between country and seniority (*p* < 0.001)—in many cases Polish nurses have more than 15 years of experience.

The greatest differences in the perception of the specificity of work in the nursing profession were observed in the case of the country characteristic. Significant differences between the opinions of Polish and German nurses occurred for 11 of 16 statements, and for 8 of these the *p*-value from the Mann-Whitney U test was below 0.001. Taking into consideration the undesirable phenomena, Polish nurses, more often than German ones, experience too much bureaucracy and high physical workload, and also dream more about taking some rest from work. At the same time, they more often agreed with the opinion that they have an influence on the way they perform their work, that they are satisfied with it and that they are suited to what they do. As for seniority, significant differences occurred in seven cases. However, these were smaller than in the case of comparisons between countries. Respondents with more than 15 years of work experience, more often than those working for a shorter time in the profession, noticed too much bureaucracy, little success at work and accompanying excessive stress, as well as the need to adapt to a large number of changes. They feel more tired, which resulted in higher average evaluations for statements regarding excessive physical workload and desire to take some rest from work. More often than people with 15 years of experience, they also agree that they are suited to the job they do. The fewest significant differences (five cases) appeared in the case of evaluations expressed by people holding management and non-management positions. The biggest difference was noted for the statement concerning high physical workload, which is less of a problem for people in management positions, who also less often thought about taking some rest from work. On the other hand, they more often assessed relationships with co-workers as stressful, and they also have a greater problem with maintaining proper work-life balance and adapting to a larger number of changes.

An analogous analysis was carried out for the remaining six questions from the questionnaire included in the article. The numerical results are presented in Table 3.

We have not found any significant differences in the assessment of the feeling of reluctance to go to work between respondents from Germany and Poland and between people with different levels of seniority. A significant difference was noted only for people in non-management and management positions—reluctance was more often felt by people who did not perform managerial functions. Polish nurses, less frequently than German nurses, declared having regular activities apart from their work and household duties, which they perform for themselves, for their own pleasure or development. The other two categories did not differentiate this phenomenon in a significant manner. Polish respondents evaluated their knowledge of occupational burnout and the ability to recognise its symptoms much more highly than German respondents. The same happened for the group of people with more than 15 years of work experience in relation to people working in the profession for a shorter period of time. The position in the workplace did not have any influence on self-assessment of knowledge of the phenomenon of occupational burnout. This is perceived as a serious problem in the healthcare sector, with the mean for the Polish sample being significantly higher than for the German. People with longer work experience seem to notice a greater risk. It is worth noting that none of the three analysed categories appeared to differentiate in the case of evaluation of the difficulty in admitting occupational burnout in the workplace. However, all differentiated when evaluating the possibilities of occupational burnout prevention in healthcare. More sceptical in this respect were respondents from Poland, people working in non-management positions and those with longer work experience.

### 3.2. Factor and Reliability Analysis for Groups of Characteristics Typical of the Nursing Profession

Exploratory factor analysis of question 1 items is presented in Table 4. Four eigenvalues are greater than one, so a four factor structure is estimated. Loadings greater than 0.45 are marked with bold red font.

The analysis of the content of the groups of questions assigned to the various factors shows that they came together because of similar content. The names that can be assigned to the four identified factors are as follows: factor 1—workload, factor 2—job satisfaction, factor 3—stressful atmosphere in the organisation, factor 4—feeling of control over one’s own work.

In order to further investigate the measurement properties, the values of the Cronbach’s alpha reliability coefficient were calculated for sub-scales developed from marked items in Table 4. Cronbach’s alpha value for Factor 1 scale (1a, 1b, 1g, 1h, 1o) equals 0.72; for Factor 2 scale (1k–1m, 1n recoded) 0.71; Factor 3 scale is almost sufficiently reliable only for the 1e–1f subscale (alpha equals 0.67); Factor 4 scale is not reliable (alpha equals 0.44). Only reliable Factors 1–3 scales were used in the next analyses.

A three factor structure was tested with the confirmatory factor analysis model (maximum likelihood estimation method) for the set question 1 items without the items (1c, 1i, 1p), which poorly correlated with the rest of items. The results of this analysis are shown in Table 5.

The low loading at 1j is an argument for not including this variable in the measurement of factor 3. The remaining factor loadings are at least 0.5 in relation to the absolute value. All the correlations between the factors are significant, with factor 1 relating to workload and factor 3 relating to the stressful atmosphere in the organisation as the most strongly correlated. Factor 2 concerning job satisfaction is negatively correlated with the others, which may indicate that satisfaction is as an individual feature of the respondents, less dependent on the external situation at work.

The evaluation of the quality of the CFA model performed with the most commonly used indicators [31] is presented in Table 6.

Comparing the obtained values from Table 6 to strict requirements given by Schreiber et al. [32], the CFA model cannot be considered as the final measurement model. Most well-matched to the data. However, these indicators show that, at the initial stage of creating such a model, a fairly good fit is achieved that could be the basis for further improvements to the measurement model. Nevertheless, due to the lack of a very good fit of the model to the data, it is reasonable to analyse both individual items, which was done point 3.1, as well as selected factors that allow us to reduce the dimension of the variables describing the work of nurses.

Finally, to recapitulate the above analysis, differences in mean values of derived reliably measured three factors are shown in Table 7 with the *p*-value from the Mann-Whitney U test to compare country, current job position and seniority categories. Polish nurses feel greater workload (Factor 1), but also greater job satisfaction (Factor 2) than German nurses. However, the difference in stressful atmosphere in the organisation (Factor 3) between countries is only on the borderline of significance (a greater feeling of stressful atmosphere among German nurses). No significant differences are observed between non-management and management positions. Nurses with seniority over 15 years feel significantly greater workload, but also greater job satisfaction (on the borderline of significance).

### 3.3. Relationship between the Factors Describing the Specificity of the Nursing Profession and the Issues of Occupational Burnout

The analysis of correlation between variables was performed using both the Pearson linear correlation coefficient and the Spearman’s rank correlation coefficient. Since the conclusions from the analysis of both types of correlations were identical, Table 8 presents the linear correlation matrix. A “Difficult to say answer” option, allowed for some questions, was always coded as missing data, a pairwise deletion of missing data was used, and a count of N was given for each value of the correlation coefficient.

Most of the observed values are significantly different from zero, but these are rather weak correlations. The strongest relationships appeared between reluctance to go to work (question 2) and Factor 1 (workload)—a positive correlation—and Factor 2 (job satisfaction)—a negative correlation, which is understandable. There is also a significant positive correlation between reluctance to go to work and stressful atmosphere in the organisation. Much lower values of the correlation coefficient occur for participation in after-work activities (question 3)—due to the coding of this variable, we interpret that the perception of work as burdening (Factor 1) is associated with less frequent participation, whereas job satisfaction (Factor 2) is associated with more frequent participation in this type of activity. The declared level of knowledge and recognition of burnout (question 4) is mainly related to higher job satisfaction, but also significantly to the perception of work as more burdening. The feeling of a general risk of burnout in the healthcare sector (question 7) is most strongly associated with the perceived workload, then with the stressful atmosphere in the organisation, and finally with lower job satisfaction. The only significantly different from zero correlation in Table 8 for the ease of admitting burnout (question 8) is observed for stressful atmosphere in the organisation resulting from the relationship with superiors and co-workers, which, however, is a weak correlation. A greater perception of burnout preventive measures (question 11) is associated with a lower perceived workload and less stressful atmosphere in the organisation, as well as higher job satisfaction.

## 4. Discussion

Referring to the research questions it is possible to state that the obtained results of the study carried out on the basis of the proprietary questionnaire showed differences in the perception of the specificity of work in the nursing profession in Poland and in Germany in relation to various aspects relating to the risk of occupational burnout. They also revealed differences in this area due to professional characteristics such as seniority or decision-making in the position held, understood as a management vs. non-management positions. The reasons for the observed significant differences may be objective financial conditions (higher remuneration in Germany and in management positions), or cultural values influencing the status of the nurse in society and the situation in the sector (very high feminisation of the profession in Poland, too few new nurses recruited resulting in aging staff). Another aspect which may also influence perception is that of selection, which means that employees suffering from burnout have less chance of longer seniority and what usually follows—taking a management position. In order to reduce the risk of occupational burnout, individual predispositions of the employee should make it possible to meet the requirements of the profession and the stress associated with it. Bearing in mind that it is not always possible to help all sick patients and enjoying the fact of helping other people, should bring satisfaction within this profession even though one’s financial expectations are not fully met. Items describing own work are grouped into 4 factors reflecting latent variables, three of which can be reliably measured—workload, job satisfaction and stressful atmosphere at work. The analysis of the relationship with the remaining questions showed that the perception of one’s job as being burdensome is most strongly associated with the reluctance to go to work, which may be a symptom of burnout. There are also quite strong relationships between workload and the perception of a higher risk of burnout in this sector, as well as poorer possibilities of preventing it. On the other hand, job satisfaction is most strongly associated with the willingness to go to work, and also with the belief that one understands the phenomenon of occupational burnout and is able to recognise it in oneself and in colleagues. A stressful atmosphere at work is most strongly associated with reluctance to go to work, as well as a feeling of a higher risk of burnout.

The obtained results confirmed those achieved by other research teams conducting studies on occupational stress or burnout symptoms in nurses. First of all, they confirmed the difficult situation in the nursing profession in both countries—the exhaustion of the staff and the lack of effective organisational solutions that could improve the situation in the future. As for the research mentioned in the introduction [16], as many as 44% of nurses from Poland and 36% from Germany indicated their intention to change their current job. The obtained results are consistent with the results of research conducted in a group of German nurses, which indicated the need to introduce preventive and intervention strategies that would focus both on the individual and on working conditions [33].

An interesting aspect of the discussion on risks in the nursing profession is the result of studies on the relationship between the sense of coherence (and its components) and patterns of professional behaviour in nurses [34]. According to this, the sense of coherence can be treated as specific personal potential for nurses, which may be of significant importance in the process of coping with stress and symptoms of burnout. Therefore, training for this professional group in soft skills, including the issues of coping with stress at work, is very important. Another way to improve the situation is to provide nurses with real-time feedback that can mitigate the negative influence of physical and mental stress on their well-being [35]. The results of research conducted in a group of nurses from Finland and the Netherlands made it possible to identify three good practices [36]: resource adequacy, management support and ensuring proper quality of care through relationships based on cooperation. Positive evaluations of the adequacy of resources and support from management were positively correlated with the nurses’ assessment of the quality of care and positive feelings about work, and negatively correlated with intentions to leave the job, the organisation or even the profession.

Referring to the skills of working under time pressure required in the nursing profession, they seem to be particularly important in the context of emergency situations. An interesting voice in the discussion are the results of research conducted on Polish and Saudi Arabian nurses, where it was found that work experience, workplace preparedness, training and experience in disaster response are key factors for the effectiveness of actions undertaken in such conditions [37,38]. In conclusion, it is worth emphasizing that the research was conducted in 2018 and 2019, i.e., in the so-called “normal”, non-pandemic conditions. The COVID-19 turmoil, which has placed a huge strain on healthcare professionals around the world, has also left its mark on the issues raised in the article. It seems that the repetition of this research after the pandemic period may provide additional information worth considering when developing prevention programs against burnout in the nursing profession.

When referring to the future of the nursing profession, the key task seems to be the improvement of the prestige of this occupation and working conditions. According to the results of the research conducted among Polish nursing students, the decision to choose the profession results mainly from practical issues related to the possibility of employment and helping others, but also from the belief that the prestige of the profession and working conditions can be improved [39]. Feeling pride in being a nurse and searching for strategies that could support nurses in their daily work have been mentioned in the results of the studies conducted among Belgian nurses [40].

## 5. Limitations

The results should be interpreted with a certain degree of caution due to the limitations of the research sample—it was not selected randomly, although we made efforts to make it representative for the analysed populations. The compared samples from two countries are not of the same size. They are characterized by a disproportion because much more data were collected in Poland than in Germany. The analysis of the relationships between characteristics and country and seniority can be biased with an undesirable relationship between them—Polish nurses have significantly longer professional experience, and therefore, they are also older.

Although it is possible to hold some reservations about the statistical correctness of the research sample, the implementation of primary research dedicated to risks in the nursing profession on research samples from two countries differing in system solutions offered numerous interesting results. The presented outcomes may encourage thinking about the effectiveness of the adopted solutions and their influence on the feeling of facing excessive stress and occupational burnout in nurses. It seems justified to conduct the research one more time on the basis of the same or similar questionnaire, especially after the pandemic period, when the situation of nurses and certainty of employment have significantly deteriorated. In the view of declining interest in nursing studies on the part of young people, many nurses retiring from work and an increasingly older population of this professional group, it seems crucial to take a closer look at how nurses perceive their occupation in the context of potential risks, find effective solutions preventing them from giving up their jobs and make the profession both more prestigious and more attractive.

## 6. Conclusions

According to the conducted research, the risk of excessive stress and burnout among nurses is noticed in both countries, but for different reasons. This is especially proven by the fact that differences in the perception of the profession among nurses from Poland and Germany occurred for 11 out of 16 statements, and for eight of these the p-value from the Mann-Whitney U test was below 0.001. It seems that Polish nurses are much more stressed and physically exhausted. This is probably due to the quite common phenomenon of working in several places, in the case of which the number of hours spent at work equals two full-time positions (the effect of very low salaries). Nevertheless, bureaucracy and lack of success or of an effective promotion path seem to be more distressing. Nurses would like to take some rest, but on the other hand they feel that they are suited to the work they do and have a greater sense of influence on how they perform it. They are also more satisfied with their work. German nurses, on the other hand, complain to a greater extent about stressful relationships with their superiors and colleagues, and they would also be more willing to change their job. In relation to the remaining questions, it is worth noting that nurses from Germany more often declared doing something for a treat, that is, having a hobby or performing activities which are not connected with their professional or household duties, which are performed only for pleasure or development, and which are often considered an effective form of prevention of occupational burnout. The research results also showed a worse evaluation of the situation by Polish nurses in the context of burnout as a serious problem for the healthcare sector and their greater scepticism concerning possible preventive measures in this regard.

## Figures and Tables

**Table 1:** Research sample—structure according to selected characteristics (N = 747).

Characteristic	Characteristic Category	Percentage of Respondents (%)
Sex	Female	91.1
	Male	8.9
Age	Less than 25 years	5.1
	25–35 years old	17.9
	36–45 years old	19.8
	46–55 years old	38.7
	56 years and more	18.5
Current job position	Non-management position	16.6
	Management position	83.4
Seniority	Less than 5 years	12.5
	5–15 years	17.9
	16–25 years	23.1
	More than 25 years	46.5

**Table 2:** Diversification of opinions concerning work in the nursing profession due to selected characteristics of the respondents.

Item	Country			Current Job Position			Seniority		
	Mean ± St. Dev.		*p*-Value	Mean ± St. Dev.		*p*-Value	Mean ± St. Dev.		*p*-Value
	DE	PL		NMP	MP		Up to 15 Years	Over 15 Years	
1a	5.64 ± 2.74	6.58 ± 2.93	***	6.38 ± 2.94	6.56 ± 2.81	ns	6.06 ± 2.76	6.60 ± 2.98	**
1b	4.60 ± 3.29	6.23 ± 3.21	***	6.23 ± 3.21	4.69 ± 3.25	***	5.63 ± 3.18	6.12 ± 3.30	*
1c	5.12 ± 2.72	5.61 ± 2.73	^‡^	5.52 ± 2.72	5.75 ± 2.77	ns	5.42 ± 2.66	5.60 ± 2.75	ns
1d	8.25 ± 3.02	9.19 ± 1.85	*	9.11 ± 1.99	8.84 ± 2.40	ns	9.00 ± 2.08	9.07 ± 2.08	ns
1e	3.97 ± 2.98	3.69 ± 3.31	ns	3.75 ± 3.28	3.88 ± 3.17	ns	3.76 ± 3.13	3.71 ± 3.32	ns
1f	4.19 ± 2.64	3.62 ± 2.98	*	3.60 ± 2.89	4.41 ± 3.05	*	3.72 ± 2.72	3.70 ± 3.02	ns
1g	5.78 ± 3.16	7.58 ± 2.91	***	7.30 ± 3.02	7.42 ± 2.92	ns	6.94 ± 2.87	7.44 ± 3.06	**
1h	5.90 ± 2.85	5.86 ± 2.92	ns	5.77 ± 2.92	6.36 ± 2.86	*	5.51 ± 2.75	6.02 ± 2.97	*
1i	4.11 ± 2.97	4.34 ± 3.27	ns	4.15 ± 3.15	4.82 ± 3.37	^‡^	4.00 ± 2.93	4.42 ± 3.33	ns
1j	3.80 ± 2.31	4.77 ± 2.76	***	4.59 ± 2.72	4.75 ± 2.63	^‡^	4.18 ± 2.52	4.83 ± 2.78	**
1k	6.90 ± 2.97	7.93 ± 2.31	***	7.76 ± 2.46	7.86 ± 2.36	ns	7.44 ± 2.53	7.94 ± 2.41	**
1l	6.37 ± 2.94	7.17 ± 2.73	**	7.12 ± 2.76	6.54 ± 2.82	*	6.89 ± 2.83	7.11 ± 2.76	ns
1m	5.97 ± 2.70	7.09 ± 2.49	***	6.88 ± 2.53	7.12 ± 2.61	ns	6.80 ± 2.45	6.98 ± 2.62	ns
1n	3.57 ± 3.29	3.22 ± 3.31	ns	3.35 ± 3.33	2.71 ± 3.01	^‡^	3.48 ± 3.07	3.15 ± 3.41	^‡^
1o	4.68 ± 3.09	6.20 ± 3.28	***	6.11 ± 3.29	5.27 ± 3.12	**	5.60 ± 3.31	6.11 ± 3.29	*
1p	5.30 ± 2.98	6.74 ± 2.68	***	6.41 ± 2.79	6.94 ± 2.64	^‡^	6.29 ± 2.68	6.62 ± 2.82	^‡^

*** *p* < 0.001, ** 0.001 ≤ *p* < 0.01, * 0.01 ≤ *p* < 0.05, ^‡^ 0.05 ≤ *p* < 0.1, ns—non-significant; DE—Germany, PL—Poland, NMP—Non-management position, MP—Management position.

**Table 3:** Differentiation of opinions in the area of occupational burnout and accompanying phenomena due to selected characteristics of the respondents.

Question	Country			Current Job Position			Seniority		
	Mean ± St. Dev.		*p*-Value	Mean ± St. Dev.		*p*-Value	Mean ± St. Dev.		*p*-Value
	DE	PL		NMP	MP		Up to 15 years	Over 15 years	
2	2.71 ± 0.94	2.70 ± 0.93	ns	2.74 ± 0.91	2.48 ± 0.95	**	2.71 ± 0.84	2.69 ± 0.96	ns
3	1.54 ± 0.65	1.69 ± 0.61	**	1.69 ± 0.62	1.59 ± 0.63	ns	1.62 ± 0.66	1.69 ± 0.60	^‡^
4	5.73 ± 2.23	7.49 ± 2.18	***	7.15 ± 2.29	7.49 ± 2.17	ns	6.63 ± 2.21	7.43 ± 2.25	***
7	6.75 ± 2.24	7.97 ± 2.40	***	7.84 ± 2.41	7.67 ± 2.39	ns	7.58 ± 2.22	7.87 ± 2.50	*
8	6.39 ± 2.57	6.10 ± 2.84	ns	6.04 ± 2.84	6.50 ± 2.63	ns	5.95 ± 2.75	6.23 ± 2.82	ns
11	1.94 ± 0.83	2.29 ± 0.92	***	2.30 ± 0.93	1.91 ± 0.77	***	2.06 ± 0.84	2.31 ± 0.93	**

*** *p* < 0.001, ** 0.001 ≤ *p* < 0.01, * 0.01 ≤ *p* < 0.05, ^‡^ 0.05 ≤ *p* < 0.1, ns—non-significant; DE—Germany, PL—Poland, NMP—Non-management position, MP—Management position.

**Table 4:** Factor loadings of exploratory factor analysis (EFA) with principal components method and varimax rotation.

Item	Factor 1	Factor 2	Factor 3	Factor 4
1a	**0.664**	0.002	0.158	0.238
1b	**0.679**	−0.081	−0.002	0.23
1c	0.116	0.095	0.079	**0.791**
1d	**0.515**	0.408	0.031	0.153
1e	0.244	−0.226	**0.681**	0.005
1f	0.139	−0.227	**0.707**	0.208
1g	**0.657**	0.162	0.244	−0.257
1h	**0.545**	0.02	0.393	−0.162
1i	−0.24	0.283	**0.469**	−0.04
1j	0.184	0.079	**0.57**	−0.023
1k	0.195	**0.769**	0.05	0.057
1l	0.05	**0.653**	−0.029	0.143
1m	−0.111	**0.79**	−0.028	0.191
1n	0.332	**−0.594**	0.137	0.087
1o	**0.62**	−0.381	0.066	0.016
1p	0.059	0.415	−0.022	**0.586**

**Table 5:** Three-factor confirmatory factor analysis (CFA) model: parameter estimates and significance.

Latent Variable	Item	Standardized Factor Loading or Correlation
Factor 1—workload	1a	0.663 ***
	1b	0.604 ***
	1g	0.540 ***
	1h	0.559 ***
	1o	0.525 ***
Factor 2—job satisfaction	1k	0.655 ***
	1l	0.542 ***
	1m	0.856 ***
	1n	−0.500 ***
Factor 3—stressful atmosphere in the organisation	1e	0.731 ***
	1f	0.680 ***
	1j	0.338 ***
Correlations between factors	r(1,2)	−0.131 **
	r(1,3)	0.575 ***
	r(2,3)	−0.240 ***

*** *p* < 0.001, ** *p* < 0.01.

**Table 6:** Characteristics and goodness-of-fit statistics for the three-factor CFA model.

Measure	Score
Chi square	406.03
degrees of freedom (df)	51
Chi square/df (minimum discrepancy)	7.96
SRMR (minimum discrepancy)	0.092
RMSEA (root mean square error of approximation)	0.098
GFI (goodness of fit index)	0.915
AGFI (adjusted goodness of fit index)	0.870
CFI (comparative fit index)	0.814
NFI (normed fit index)	0.794

**Table 7:** Differentiation of opinions concerning work in the nursing profession due to selected factors.

Factor	Country			Current Job Position			Seniority		
	Mean ± St. Dev.		*p*-Value	Mean ± St. Dev.		*p*-Value	Mean ± St. Dev.		*p*-Value
	DE	PL		NMP	MP		Up to 15 Years	Over 15 Years	
1	5.32 ± 2.11	6.49 ± 2.08	***	6.36 ± 2.13	6.09 ± 1.99	ns	5.95 ± 2.02	6.46 ± 2.14	***
2	6.42 ± 2.19	7.25 ± 1.99	***	7.11 ± 2.05	7.22 ± 1.94	ns	6.93 ± 2.08	7.22 ± 2.03	^‡^
3	4.08 ± 2.43	3.66 ± 2.75	^‡^	3.68 ± 2.67	4.17 ± 2.79	ns	3.74 ± 2.57	3.71 ± 2.76	ns

*** *p* < 0.001, ** 0.001 ≤ *p* < 0.01, * 0.01 ≤ *p* < 0.05, ^‡^ 0.05 ≤ *p* < 0.1, ns—non-significant; DE—Germany, PL—Poland, NMP—Non-management position, MP—Management position.

**Table 8:** Pearson’s linear correlations between the factors describing the specificity of the nursing profession and the issues of occupational burnout.

Question	Factor 1	Factor 2	Factor 3
2	0.4484	−0.487	0.2873
	N = 743	N = 741	N = 738
	***	***	***
3	0.1347	−0.1415	0.0523
	N = 737	N = 735	N = 732
	***	***	ns
4	0.0978	0.1939	−0.0149
	N = 727	N = 725	N = 723
	**	***	ns
7	0.297	−0.0995	0.1666
	N = 691	N = 689	N = 687
	***	**	***
8	0.0553	0.0743	0.0794
	N = 658	N = 657	N = 655
	ns	ns	*
11	0.1602	−0.1221	0.132
	N = 581	N = 581	N = 579
	***	**	**

*** *p* < 0.001, ** 0.001 ≤ *p* < 0.01, * 0.01 ≤ *p* < 0.05, ns—non-significant.

## Data Availability

The dataset presented in the study is available upon request from the corresponding author.

## Appendix A

**Table A1:** General questions about work: Please assess to what extent each of the questions describes your current job.

No.	Item	Response Variants and Coding
1a	High emotional stress	The scale of 0 to 10, where 0—definitely inadequate, 10—definitely adequate
1b	Great physical strain	
1c	I am mostly responsible for my work results	
1d	My work involves contact with others	
1e	A stressful relationship with a supervisor	
1f	Stressful relationships with coworkers	
1g	Too much bureaucracy	
1h	A dynamic environment/a necessity to adapt	
1i	An adequate remuneration for work	
1j	Little success at work	
1k	I feel that my work suits me	
1l	I maintain a work-life balance	
1m	I am satisfied with my work	
1n	I would like to change my job	
1o	I wish I could take some time off work	
1p	I can influence the way I do my work	

**Table A2:** The remaining analysed questions.

No	Question	Response Variants and Coding	
2	How often do you feel like not going to work, when thinking about your current job?	Never	1
		Very rarely	2
		Occasionally	3
		Often	4
		Constantly	5
3	Do you take part in any leisure or personal development activities, other than your professional and domestic duties?	Yes, regularly	1
		Yes, although sporadically	2
		No	3
4 ^1^	Please think about “burnout” for a moment. How would you describe your level of understanding of this phenomenon? Please describe to what extent you agree with the following statements. a. I have sufficient understanding of burnout b. I could recognize the symptoms of burnout if they affect me personally c. I am able to tell whether somebody else shows the symptoms of burnout	The scale of 0 to 10 where 0—I completely disagree, 10—I completely agree	
7	When thinking about your industry, could you describe how serious the problem of burnout is?	The scale of 0 to 10 where 0—it is not a problem, 10—it is a serious problem	
8	When thinking about your industry—how easy would it be to admit to being burn-out at a workplace if somebody’s position were similar to yours?	The scale of 0 to 10 where 0—very easy, 10—very difficult	
11	Do you think the prevention of burnout is possible in your industries?	Definitely yes	1
		Rather yes	2
		Rather no	3
		Definitely no	4
		Difficult to say	5

^1^ In the case of question 4, we combined responses to 3 statements (a, b, c) due to their strong correlation and analysed them together as the mean of the responses to the 3 statements given with Cronbach’s alpha value equal to 0.86.
